# A Laboratory Test Setup for *in Situ* Measurements of the Dielectric Properties of Catalyst Powder Samples under Reaction Conditions by Microwave Cavity Perturbation: Set up and Initial Tests

**DOI:** 10.3390/s140916856

**Published:** 2014-09-10

**Authors:** Markus Dietrich, Dieter Rauch, Adrian Porch, Ralf Moos

**Affiliations:** 1 Department of Functional Materials, University of Bayreuth, 95440 Bayreuth, Germany; E-Mails: Dieter.Rauch@uni-bayreuth.de (D.R.); Ralf.Moos@Uni-Bayreuth.de (R.M.); 2 School of Engineering, Cardiff University, Cardiff, CF24 3AA, Wales, UK; E-Mail: PorchA@cardiff.ac.uk

**Keywords:** ammonia SCR, ammonia storage, H-ZSM-5, cavity perturbation, complex permittivity, in operando, radio frequency

## Abstract

The catalytic behavior of zeolite catalysts for the ammonia-based selective catalytic reduction (SCR) of nitrogen oxides (NO_X_) depends strongly on the type of zeolite material. An essential precondition for SCR is a previous ammonia gas adsorption that occurs on acidic sites of the zeolite. In order to understand and develop SCR active materials, it is crucial to know the amount of sorbed ammonia under reaction conditions. To support classical temperature-programmed desorption (TPD) experiments, a correlation of the dielectric properties with the catalytic properties and the ammonia sorption under reaction conditions appears promising. In this work, a laboratory test setup, which enables direct measurements of the dielectric properties of catalytic powder samples under a defined gas atmosphere and temperature by microwave cavity perturbation, has been developed. Based on previous investigations and computational simulations, a resonator cavity and a heating system were designed, installed and characterized. The resonator cavity is designed to operate in its TM_010_ mode at 1.2 GHz. The first measurement of the ammonia loading of an H-ZSM-5 zeolite confirmed the operating performance of the test setup at constant temperatures of up to 300 °C. It showed how both real and imaginary parts of the relative complex permittivity are strongly correlated with the mass of stored ammonia.

## 1. Introduction

The reduction of emissions like nitrogen oxides (NO_X_) from the exhaust gas of combustion engines are a continuous factor in forcing automotive manufacturers to improve the efficiency of their after-treatment systems for exhaust gases. Especially for light and heavy duty diesel engines, which are operated leanly, the ammonia-based selective catalytic reduction (SCR) has become the major NO_X_ control strategy to meet emission standards like the upcoming Euro 6 [1]. In this process, an aqueous urea solution (AdBlue) is injected into the hot exhaust, where it decomposes thermally to the reducing agent ammonia (NH_3_). Ammonia reduces NO_X_ at the SCR-catalyst selectively to nitrogen (N_2_) and water (H_2_O). There are two main SCR reactions besides several subordinate reactions: the standard SCR reaction (1) implying a 1:1-stoichiometry for NH_3_ and NO, and the fast SCR reaction (2) with equimolar amounts of NO and NO_2_ [2].

(1) $$4\;{\text{NH}}_{3}+4\;\text{NO}+{\text{O}}_{2}\to 4\;{\text{N}}_{2}+{6\;\text{H}}_{2}\text{O}$$

(2) $$4\;{\text{NH}}_{3}+2\;\text{NO}+{2\;\text{NO}}_{2}\to 4\;{\text{N}}_{2}+6\;{\text{H}}_{2}\text{O}$$

The SCR catalyst adsorbs ammonia to buffer changes of flow and temperature of the exhaust gas in order to secure a permanent NO_X_ conversion [2]. Metal oxides like V_2_O_5_-WO_3_/TiO_2_ (VWT) are established as SCR-catalysts, but as a consequence of their classification as being toxic and dangerous for the environment, zeolites with active components like iron (Fe) and copper (Cu) receive more attention [3]. Ammonia storage capacity and catalytic activity of zeolite SCR catalysts depend on the strength of their acid sites (Lewis and Brønsted sites). Both number and strength can be determined by temperature-programmed desorption (TPD) of ammonia, but a direct differentiation between Lewis and Brønsted sites is still not possible [4]. Therefore, the ability to measure the dielectric properties under reaction conditions during ammonia storage, and to correlate them with the catalytic behavior of the materials *in situ*, offer new opportunities to identify acidic sites and to optimize the catalyst material in general. At this point, the contactless cavity perturbation method, which uses microwaves and a metal cavity resonator, holds much promise. Recently, a similar approach has been suggested and serial-type catalyst devices (as applied in automotive exhaust gas after-treatment) have been studied [5]. Both the catalytic converter and metal canning (resonator wall) have volumes of about 1.5 to 2 liters. Since the sample occupies most of the cavity volume, these systems are suitable for real-world applications but not to characterize materials owing to their very large perturbation on the cavity space. Instead, they are intended to detect the status of full-sized exhaust gas after treatment devices during operation on the road. Typical applications are the determination of the oxygen loading of three-way catalytic converters [5–8], or the soot loading [9,10] or the ash loading [11] of full-sized diesel particulate filters. The storage degree of NO in lean NO_X_ traps [12,13] and the ammonia loading on SCR catalyst devices have also been successfully monitored [14,15].

The so-called small object cavity perturbation, however, in which the samples occupy only a small fraction of the cavity volume, enables highly accurate measurements of the complex relative permittivity of material samples [16–18]. In an earlier approach, the conductivity of metal ion exchanged zeolites has been observed [19,20] in this way.

In order to determine catalyst material properties *in operando*, a laboratory test setup to measure the dielectric properties of catalyst powder samples under reaction conditions by microwave cavity perturbation was developed in this study. It enables direct measurements of the complex dielectric materials properties of catalytic powder samples undergoing gas storage and catalytic reactions in a defined gas atmosphere, with gas analyzers up- and downstream of the catalyst sample. It operates in a temperature range from room temperature (where usually no reactions occur) to 300 °C.

## 2. Methods: Set up, Characterization and Fundamentals of the Test Setup

**Figure 1.**
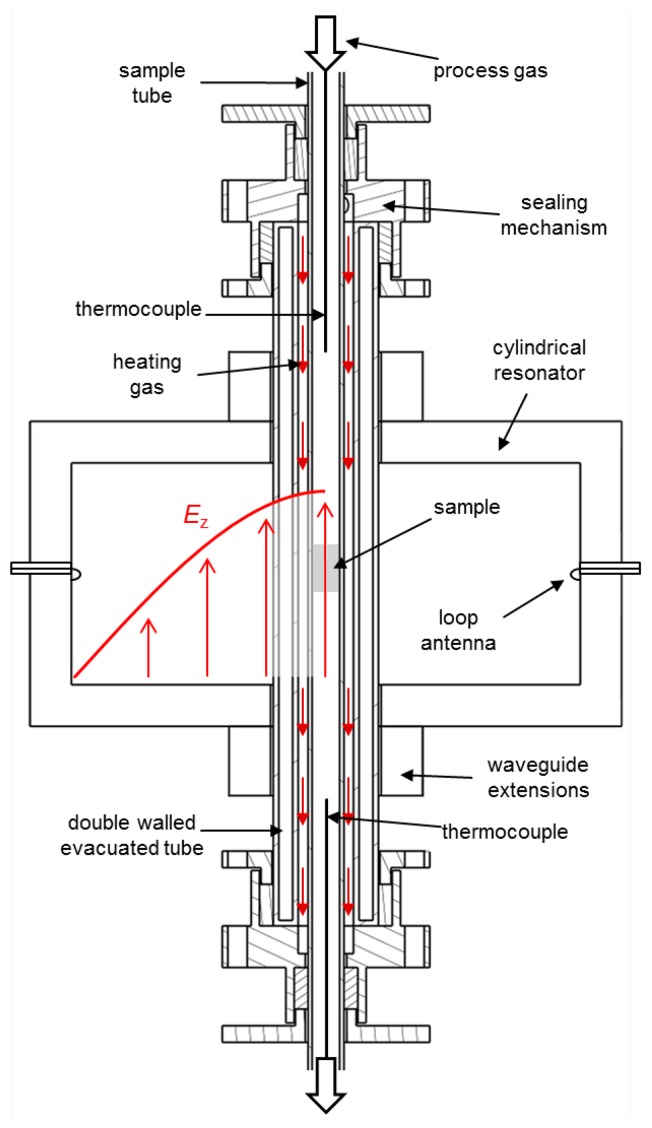
Schematic setup of the developed test bench including the sample tube, the double-walled and evacuated tube, the sealing mechanism, the resonator with waveguide extensions, loop antennas and two thermocouples. The gas heater, the Fourier transform infrared (FTIR) gas analyzer and the network analyzer (all external components) are not shown.

The schematic setup of the laboratory test setup developed in this work is illustrated in Figure 1. In microwave cavity perturbation it is desirable for the sample to dominate the microwave losses, rather than other component parts. For that reason, the resonator was fabricated from aluminum, a high conductivity metal, and all sample tubes were made from low-loss quartz. For the fixed-bed catalyst samples, a porous frit in the sample tube with an inner diameter of 10 mm was provided. The process gas entered the sample tube and was preheated to prevent the condensation of gas components and ammonia nitrate formation in the feed lines. A heated line led the process gas to the Fourier transform infrared (FTIR) spectrometer for outlet gas analysis. A double-walled (and permanently evacuated) second tube was placed around the sample tube to minimize thermal conduction. It had an inner diameter of 20 mm, an outer diameter of 38 mm and an evacuated space between the walls of width 5 mm. Within this gap, a spiral-shaped gas stream of hot air was flowed around the sample tube to heat up both the process gas and the powder catalyst sample. For the purpose of avoiding the need of permanent attachment of the quartz tubes, a sealing mechanism, which used a packing gland with a sealing cord, was developed and tested successfully. This steel-made sealing mechanism was also necessary as a connection to the gas heating system.

Unlike [17], who use the TM_110_ mode, the cavity was designed for analyzing the TM_010_ mode. The electric field distribution of the TM_010_ mode is also illustrated in Figure 1. The TM_010_ mode of a cylindrical cavity is chosen for dielectric property measurement owing to the high, uniform electric field near the cavity axis. The electric field in these modes is directed parallel to the axis, thus parallel to the axis of the quartz sample tubes, resulting in only minor modifications of the local electric field by the presence of these tubes.

The theoretical values of the resonant frequency *f*_0_ and unloaded quality factor *Q*_0_ of the TM_010_ mode of an empty cylindrical cavity can be calculated analytically by solving the Helmholtz equation subject to the boundary conditions imposed by the metal walls, giving

(3) $$f_{0}=\frac{c}{2\pi a}p_{01}$$

(4) $$Q_{0}=\frac{1}{\delta }\frac{aL}{a+L}$$

where *a* is the cavity inner radius, *L* the inner length, *c* is the speed of light in vacuum and *δ* is the skin depth of the metal walls at the TM_010_ resonant frequency; *p*_01_ = 2.405 is the first zero of the Bessel function *J*_0_(*x*).

The sample was placed in the uniform electric field maximum on the axis of the cylindrical cavity for maximum sensitivity. In accordance to [18], the electric field shows no dependence along the gas flow direction for this resonator design operating in the TM_010_ mode. Therefore, the results are unaffected by the exact sample position along the flow direction. For reliable data, it is necessary to ensure that the TM_010_ mode is always free of interactions with any higher mode and so the aluminum cavity was designed to have an inner diameter of 184 mm and a height of 80 mm. These are both large enough to ensure that sample volumes were small by comparison for cavity perturbation theory to hold. The length to diameter aspect ratio is small enough to ensure that modes such as TE_111_ are much higher in frequency than TM_010_, so there was no mode interference. The TM_010_ mode was excited inductively by means of two loop-terminated coaxial lines around the cavity's perimeter. The cavity was measured in transmission mode using a vector network analyzer (Anritsu VNA Master MS2028B). A non-linear least-squares curve fitting to a Lorentzian response was used to determine resonant frequencies and loaded quality factors (denoted as *Q*_L_). We removed the effects of cavity couplings by converting *Q*_L_ into the unloaded quality factor *Q*_0_ in each case. The cavity couplings were carefully adjusted so that they were equal (*i.e.*, symmetric) and so the conversion was made using the simple formula *Q*_0_ = *Q*_L_(1−10^−^*^IL^*^/2^°)), where *IL* is the insertion loss (*i.e.*, transmitted power) at resonance, measured in dB.

To prevent perturbations to the resonator from the environment and prevent energy from the cavity being lost through radiation, two cylindrical waveguide axial extensions were provided for the holes for the two quartz tubes. These acted as waveguides well below cut-off for the frequency of the TM_010_ mode, whose length was chosen to equal to their inner diameter [21]. Their successful function has been tested experimentally.

**Figure 2.**
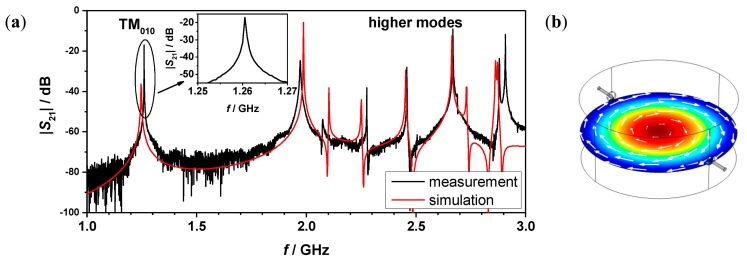
Absolute value of the transmission parameter *S*_21_ between 1 and 3 GHz: measurement (black) and simulation (red) (**a**). The simulated electric (red: High and blue: Zero) and magnetic (white arrows) field distribution of the TM_010_ mode (**b**).

A comparison of a recorded transmission spectrum (|*S*_21_|) in the frequency range from 1 to 3 GHz with a simulated spectrum (COMSOL Multiphysics, RF module) of the empty cavity is shown in Figure 2a. The skin effect of the aluminum cavity was considered by using the impedance boundary condition in the simulation model. It is apparent that the cavity performs as expected. The TM_010_ mode occurs at 1.26 GHz, which is 750 MHz lower than any higher mode, as has been designed. The unloaded quality factor, *Q*_0_, of the empty cavity for the TM_010_ mode has been measured to be 14370, which is high enough to enable highly accurate measurements based on cavity perturbation theory. Additionally the simulated electric and magnetic field distribution of the TM_010_ mode is displayed in Figure 2b.

**Figure 3.**
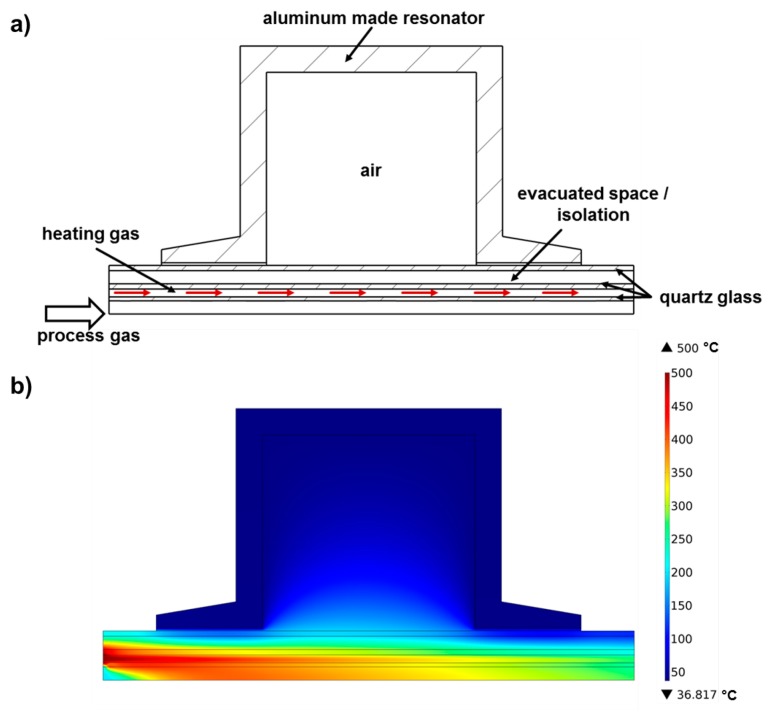
Thermal simulation model of the heating mechanism: Scheme of the model (**a**) and temperature distribution of a simulation (**b**).

To develop the sample heating system, a simulation model (COMSOL Multiphysics, conjugate heat transfer module) of the resonator and the glass tubes, considering thermal radiation and convective cooling, was established for this purpose (Figure 3a). To minimize the computing efforts, a two-dimensional rotational symmetric model was chosen and laminar flow was assumed for all gas streams. Figure 3b shows exemplarily the simulated temperature distribution for a process gas flow of 1 L/min preheated to 200 °C, a heating gas flow of 30 L/min, and a heating gas inlet temperature of 500 °C. The large temperature gradient apparent within the process gas can be explained by the consideration of thermal radiation and the non-ideal vacuum isolation in the simulation. Inside the fixed bed sample, a temperature gradient of up to 50 °C was simulated over a length of 30 mm.

**Figure 4.**
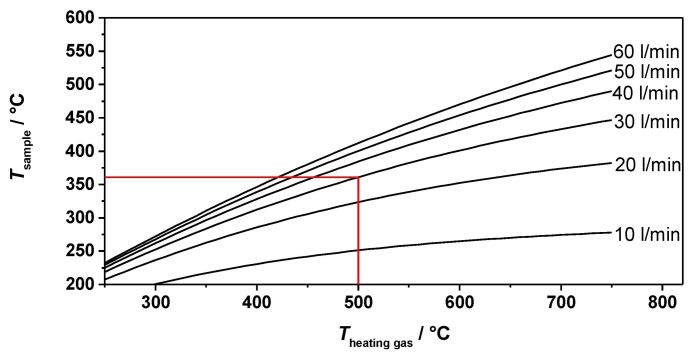
Results of the heating simulation: temperature of the sample as a function of the heating gas inlet temperature and the heating gas flow. The red line indicates the expected temperature using the available heating system.

Figure 4 displays the simulated temperature in the middle of the sample and its dependence on the heating gas inlet temperature and the heating gas flow. The process gas flow was again set to 1 L/min. The heating system available for the first test allowed a maximum heating gas temperature of almost 500 °C at a constant gas flow of 30 L/min. According to the simulations, a maximum sample temperature of about 360 °C was expected (red line in Figure 4).

**Figure 5.**
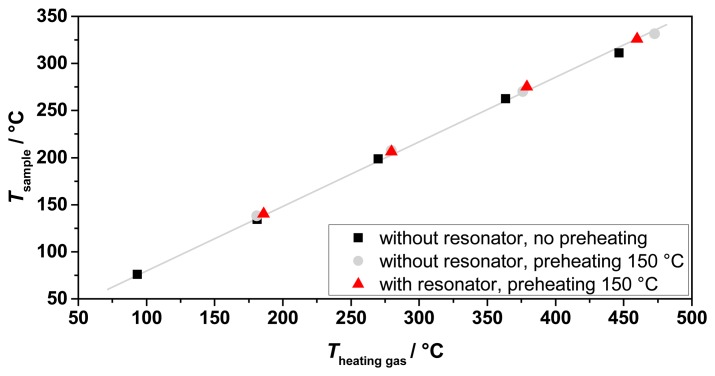
Results of the heating tests with and without resonator and preheating.

**Figure 6.**
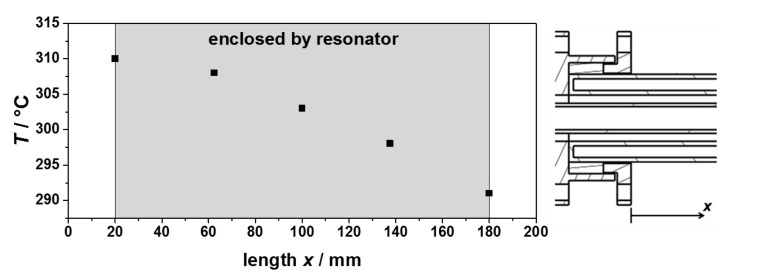
Temperature distribution inside the sample tube along its axis at a heating gas temperature of 430 °C. The sample tube is enclosed by the resonator in the gray area.

To validate the expected process gas temperatures and to characterize the heating system, several tests were made. Their results are shown in Figure 5. Tests with and without the resonator and preheating have been performed. In all tests, an almost linear relation between the sample temperature achieved and the heating gas inlet temperature has been observed, in accordance with the simulations. No influence was observed of the presence of the resonator and the preheating to the obtained process gas temperatures. Hence, the sample temperature is mostly dependent of the heating gas inlet temperature and the heating gas flow. Recording the temperature distribution inside the sample tube along its axis showed an almost linear temperature profile (Figure 6). Thus, the temperature determination of the sample was simplified by averaging the upstream and downstream temperatures of the process gas at the waveguide attachment openings, in order not to disturb the microwave electric field inside the resonator by a thermocouple. The measured temperature gradient along the tube turned out to be smaller than simulated. The temperature gradient inside the fixed bed over a sample with an assumed length of 40 mm was smaller than 5 °C.

In parallel with the heating tests with resonator, the TM_010_ mode has been tracked (results in Table 1). With an increasing heating gas temperature, a frequency shift to lower frequencies appeared. At a heating gas temperature of 460 °C, the resonance frequency shifted by 1.19 MHz from 1.17898 GHz at room temperature to 1.17779 GHz. The unloaded quality factor of the resonator with inserted glass tubes changed from 9715 to 9207. Assuming a linear thermal expansion of the cavity as the main reason for the frequency shift, a resonator temperature of almost 70 °C for 460 °C heating gas temperature would be obtained from Equation (3). Consequently, the warming of the resonator would cause major errors in measurements with variable temperatures, *i.e.*, temperature-programmed desorption (TPD) measurements. Additionally, high resonator temperatures could damage the antennas. In order to avoid this error source and possible damages, the first version of the test setup was restricted to experiments at sample temperatures of up to 300 °C.

After characterizing the heating behavior and the cross sensitivities to the microwave measurements, the test setup was ready for a first measurement. We converted the cavity measurements into dielectric properties of the materials using first order cavity perturbation theory, which we next describe. The fundamental interaction of a dielectric material with a microwave electric field is quantified by the complex relative permittivity *ε* = *ε*_1_ − j*ε*_2_ of the material, where *ε*_1_ (or, more properly, *ε*_1_ − 1) quantifies the polarization of the material in an applied electric field, whilst *ε*_2_ quantifies the dielectric loss. First order cavity perturbation theory relates the changes in the resonator parameters to the dielectric properties of a sample inserted into the electric field antinode of the cavity. If insertion of a small sample into a cavity found to reduce the resonant frequency from *f*_0_ to *f*_s_, and reduce the unloaded quality factor from *Q*_0_ to *Q*_s_, these changes are related to the complex permittivity of the sample by the approximate equations

(5) $$\frac{f_{0}-f_{s}}{f_{0}}\approx \left({ɛ}_{1}-1\right)\frac{V_{\text{s}}}{2V_{\text{eff}}}$$

(6) $$\frac{1}{Q_{s}}-\frac{1}{Q_{0}}=\Delta \left(\frac{1}{Q}\right)\approx {ɛ}_{2}\frac{V_{\text{s}}}{V_{\text{eff}}}$$

where *V*_s_ is the sample volume and *V*_eff_ is the mode volume of the cavity, which can be calculated analytically from the distribution of the electric field in the cavity as *V*_eff_ = π*a*^2^*L J*_1_^2^(*p*_01_), *i.e.*, about 26.9% of the actual internal volume of the cylindrical cavity.

Since *V*_eff_ plays a crucial role in determining dielectric properties from the resonator measurements, it is important to be able to experimentally verify its value for any cavity using the perturbation technique. Therefore, it can also be found by the calibration measurement of a known material of known geometry. We use stainless steel spheres, since a metal sphere will develop a known electric dipole moment *p* = 4πε_0_*b*^3^*E*_0_ (where *b* is the sphere radius) for which (*f*_0_ − *f*_s_)/*f*_0_ ≈ 2π*b*^3^/*V*_eff_ and so *V*_eff_ is then easy to calculate experimentally.

Before moving on to the first measurement, we note that complex permittivity values extracted from this analysis are effective values *ε*_eff_ = *ε*_1,eff_ – j*ε*_2,eff_ since the samples are powders, consisting of mostly air and about 30%–40% by volume solid particles. An effective medium approach is needed to extract the intrinsic permittivity *ε* of the actual powder grains, but this level of sophistication is not required if the cavity perturbation technique is simply to be used as a means of establishing the condition of a powder sample, as in this example with respect to the amount of ammonia absorbed by a powder zeolite catalyst.

## 3. Results and Discussion of First Measurements of the Test Setup

The sample under investigation was an H-ZSM-5 zeolite (SiO_2_/AlO_2_ = 27). The powder was kindly provided by Clariant International Ltd. For the measurement, a mass of 0.886 g of zeolite powder was used and the sample volume was determined to be 0.4067 cm^3^ by a gas pycnometer. For the analysis of the microwave data, the volume occupied by the sample and not the volume of the whole fixed bed was used.

**Figure 7.**
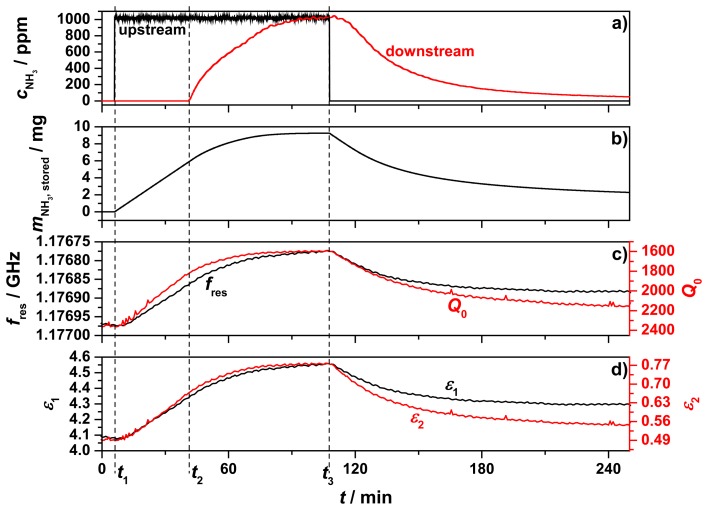
First measurement with the test setup: Upstream (inlet) and downstream (outlet) ammonia concentration (**a**), calculated stored amount of ammonia (**b**), resonance frequency and unloaded quality factor of the TM_010_ mode (**c**) and calculated effective values of the complex permittivity (**d**).

The results of the first measurement using the test setup are displayed in Figure 7. The chosen total process gas flow was reduced to 210 mL/min due to the unexpected high backpressure of the powder sample and the porous frit. The process gas that passed through the sample tube was preheated to 200 °C and initially consisted of 5% oxygen (by volume) in nitrogen. The sample temperature was kept constant for over four hours at 205 °C. In Figure 7a, the ammonia concentrations within the upstream inlet (black) and downstream outlet (red) are displayed. At *t*_1_, 1000 ppm ammonia was admixed and after 40 min (*t*_2_) ammonia breakthrough appeared. About 60 min later, the sample was fully loaded with ammonia, reflected by the identical ammonia concentrations detected upstream and downstream of the zeolite sample. Then, at *t*_3_, the ammonia feed was turned off. In the following 2.5 h until the end of the measurement, a slow desorption of ammonia occurred, indicated by the decreasing downstream ammonia concentration. From the admixed upstream ammonia concentration and the measured downstream concentration of NH_3_, the amount of stored ammonia was calculated (Figure 7b). Since neither ammonia oxidation to N_2_ and water should occur [22], nor oxidation products like N_2_O, NO, or NO_2_ were found, the timely integration of the difference between upstream and downstream ammonia concentration yielded the stored amount of ammonia in the sample. Ammonia desorption of H‐type zeolites at 200 °C is difficult and takes a long time [23]. Even two hours after the ammonia feed was stopped, it was observed that 2 mg of ammonia were still stored at the end of the measurement.

The resonance frequency, *f*_res_, and the unloaded quality factor, *Q*_0_, of the TM_010_ mode are displayed in Figure 7c. Both parameters mirrored the ammonia storage qualitatively, but with a slightly different behavior. The maximum change of *f*_res_ was 180.2 kHz and *Q*_0_ changed from 2300 to 1600 while storing ammonia. These changes can be understood by the highly polar nature of the ammonia molecule. Increased ammonia concentration in a solid sample would be expected to increase its real permittivity (giving rise to a decreased resonant frequency) and also increase its imaginary permittivity (giving rise to a reduction in quality factor). The complex relative permittivity of the sample calculated from the changes of *f*_res_ and *Q*_0_ (using Equations (5) and (6)) is shown in Figure 7d. We denote these as effective values of permittivity for the powder samples, and did not use effective medium theory to correct for volume fraction and shapes of the powder particles since all of the important information is contained in these effective values. The imaginary part of the permittivity, *ε*_2_ (red line), showed a stronger response to the stored ammonia and fitted better to the ammonia storage curve than the permittivity *ε*_1_ (black line) itself, especially during desorption. *ε*_1_ changed its value while loading from 4.12 to 4.55 and *ε*_2_ from 0.505 to 0.778 at 205 °C. All of this occurred at a measurement frequency of 1.177 GHz.

Investigations on H-ZSM-5 zeolites under ammonia storage and desorption have already been made by impedance spectroscopy in a frequency range of 0.1 Hz to 1 MHz [24,25]. Though the measurement frequency of the new setup was more than three to six decades higher, the ammonia storage affected the dielectric properties of the zeolite similarly.

The next steps with the setup will be the implementation of a more powerful heater system and an active cooling for the resonator to enable higher and variable sample temperatures for TPD measurements. With these modifications, the test setup will be a powerful tool in characterization and differentiation of catalytic powder samples in their storage behavior and catalytic properties.

## 4. Conclusions

In this work, a test setup for the dielectric characterization at elevated temperatures of catalytic powder samples by the microwave cavity perturbation has been developed. Based on previous investigations and computational simulations, a resonator cavity was designed, installed and successfully tested. A hot air heating system was simulated and also successfully implemented. In both cases, simulations and measurements agreed quite well. To implement the hot air heating mechanism, an appropriate sealing mechanism had to be developed. Its function was proven in first heating tests. An unintended warming of the resonator (measured by the associated shift of its resonance frequency) restricted measurements to constant temperatures of up to 300 °C. Thus, our target to perform TPD measurements has not been achieved yet. The first measurement of the ammonia loading of an H-ZSM-5 zeolite at a constant temperature, however, confirmed the operating performance of the test setup. The ammonia loading could be monitored by using measurements of both the resonant frequency and the quality factor of the zeolite filled resonator. The resulting values of complex permittivity *ε*_1_ and *ε*_2_ exhibited different responses to the ammonia adsorption, especially during desorption. These differences could give more information about the type and the strength of acid sites and merit further investigations.

## Figures and Tables

**Table 1.** Influence of the heating on the microwave measurement: heating gas temperature, frequency shift and quality factor. The resonator temperature is calculated from the frequency shift.

**Measurement**					**Calculation**

*T*_heating gas_/°C	*f*_res_/GHz	Δ*f*/kHz	Δ*f*/*f*_res, 25 °C_/%	*Q*_0_	*T*_resonator_/°C
25	1.1789784	0	0	9715	25
186	1.1788015	−176.9	−0.0150	9508	33
280	1.1785603	−418.2	−0.0355	9359	40
380	1.1781235	−854.9	−0.0725	9365	57
460	1.1777885	−1190	−0.1009	9207	69
